# Hax-1 is rapidly degraded by the proteasome dependent on its PEST sequence

**DOI:** 10.1186/1471-2121-13-20

**Published:** 2012-07-24

**Authors:** Bin Li, Qingsong Hu, Ranjie Xu, Haigang Ren, Erkang Fei, Dong Chen, Guanghui Wang

**Affiliations:** 1 Laboratory of Molecular Neuropathology, Department of Pharmacology, Soochow University College of Pharmaceutical Sciences, Suzhou, Jiangsu, 201203, People's Republic of China; 2 Laboratory of Molecular Neuropathology and Key Laboratory of Brain Function and Diseases, School of Life Sciences, University of Science & Technology of China, Chinese Academy of Sciences, Hefei, Anhui, 230027, People's Republic of China

**Keywords:** Hax-1, Proteasome, Ubiquitin, PEST sequence, Bcl-2 family protein

## Abstract

**Background:**

HS-1-associated protein X-1 (Hax-1), is a multifunctional protein that has sequence homology to Bcl-2 family members. *HAX-1* knockout animals reveal that it plays an essential protective role in the central nervous system against various stresses. Homozygous mutations in the *HAX-1* gene are associated with autosomal recessive forms of severe congenital neutropenia along with neurological symptoms. The protein level of Hax-1 has been shown to be regulated by cellular protease cleavage or by transcriptional suppression upon stimulation.

**Results:**

Here, we report a novel post-translational mechanism for regulation of Hax-1 levels in mammalian cells. We identified that PEST sequence, a sequence rich in proline, glutamic acid, serine and threonine, is responsible for its poly-ubiquitination and rapid degradation. Hax-1 is conjugated by K48-linked ubiquitin chains and undergoes a fast turnover by the proteasome system. A deletion mutant of Hax-1 that lacks the PEST sequence is more resistant to the proteasomal degradation and exerts more protective effects against apoptotic stimuli than wild type Hax-1.

**Conclusion:**

Our data indicate that Hax-1 is a short-lived protein and that its PEST sequence dependent fast degradation by the proteasome may contribute to the rapid cellular responses upon different stimulations.

## Background

HS-1-associated protein X-1, Hax-1, is a 35 kDa protein with two Bcl-2 homology (BH) domains that was identified in a yeast two hybrid screen where it was found to interact with HS-1, a Src kinase substrate [1]. Hax-1 is ubiquitously expressed in most tissues and is reported to be localized in mitochondria as well as the endoplasmic reticulum (ER) and nuclear membrane [1-3]. Mutations identified in the human *HAX-1* gene have been shown to cause neutropenia and neurodevelopmental abnormalities [4-6]. Knockout *HAX-1* mice show increased apoptosis of neurons and postnatal lethality. [7]. Hax-1 is a multifunctional protein that plays roles in calcium homeostasis [8], cell migration [9] and apoptotic regulation [10,11]. It was reported that Hax-1 protects cells against various stimuli and has been shown to interact with a number of cellular and viral proteins to suppress their pro-death properties [12-15]. In addition, Hax-1 has been found to be up-regulated in breast cancer, lung cancer and melanoma [16], suggesting that it also has a role in oncogenesis.

A PEST sequence is a peptide sequence which is rich in proline (P), glutamic acid (E), serine (S), and threonine (T). It is known that the PEST sequence functions as a proteolytic signal to target proteins for degradation resulting in short intracellular half lives [17]. For example, the PEST sequence of NF-kappa B is responsible for its cleavage by calpain [18]. It was reported that c-myc, a protein with a PEST sequence, has a half-life shorter than one hour [17]. Notch 1, another short-lived protein, is ubiquitinated by an E3 ligase sel-10 and degraded by the proteasome dependent on its PEST sequence [19,20].

Hax-1 was predicted to contain a PEST sequence (aa 104–117) [1], however, it is still unknown whether this PEST sequence effects its turnover rate. In this study, we investigated the stability of Hax-1 in different cells and explored the role of the PEST sequence in its degradation and biological function.

## Results

### Rapid degradation of Hax-1

**Figure 1:**
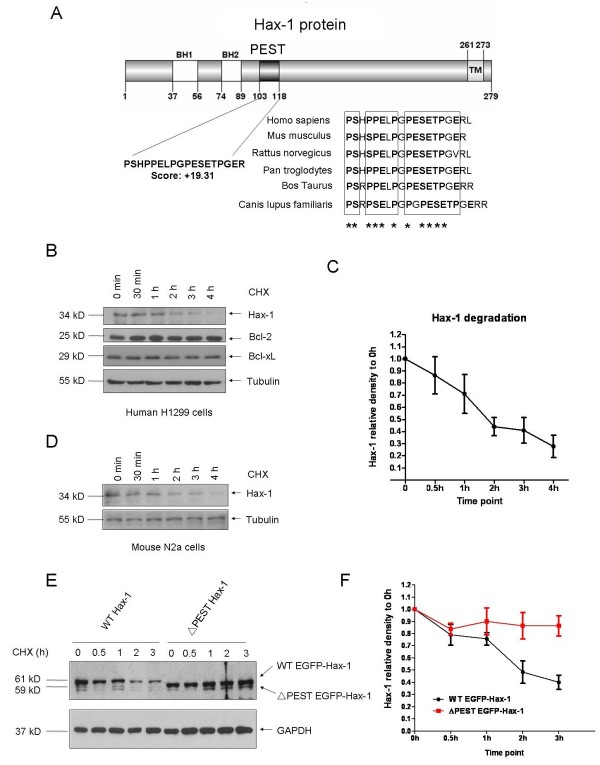
**Rapid degradation of Hax-1 is dependent on its PEST sequence. A**. Schematic representation of a PEST sequence in Hax-1 protein. The PEST sequence was identified using Pestfind service on “emboss.bioinformatics.nl/cgi-bin/emboss/pestfind”. The PEST sequence in Hax-1 is conserved among different mammals. **B**. Chase-time experiment of Hax-1 and other Bcl-2 proteins. H1299 cells treated with CHX (100 ug/ml) for different time points were harvested for immunoblot analysis using indicated antibodies. **C**. Data from three independent experiments in B were quantified. **D**. Similar experiments as B were carried out using mouse N2a cells. **E**. An EGFP-tagged WT Hax-1 or ΔPEST Hax-1 was transiently transfected into H1299 cells. Forty-eight hours later, CHX chase experiments were carried out. **F**. Quantitative analysis of data from E with three independent experiments.

In addition to its BH domains and a trans-membrane domain, Hax-1 has a PEST sequence [1]. The PEST region in Hax-1 is highly conserved in mammalian animals (Figure 1A). We tested the degradation profile of Hax-1 using a cycloheximide (CHX) chase experiment in both human lung cancer cell line H1299 and mouse neuroblastoma cell line N2a. Hax-1 was found to have a much shorter half-life than other two pro-survival Bcl-2 family proteins, Bcl-2 and Bcl-xL (Figure 1B-D), suggesting that the Hax-1 protein is unstable and is rapidly degraded.

### PEST sequence-dependent degradation of Hax-1

We next tested whether the PEST sequence in Hax-1 is responsible for its rapid degradation. A deletion mutant of Hax-1 was constructed in which the PEST sequence (aa 103–118) was deleted. The CHX chase experiments showed that the ΔPEST Hax-1 level remained largely unchanged up to 3 hours, whereas WT Hax-1 level rapidly decreased to < 50 % within 3 hours (Figure 1E and F), suggesting that the PEST sequence in Hax-1 is necessary for its rapid degradation.

### Degradation of Hax-1 by the ubiquitin-proteasome pathway

**Figure 2:**
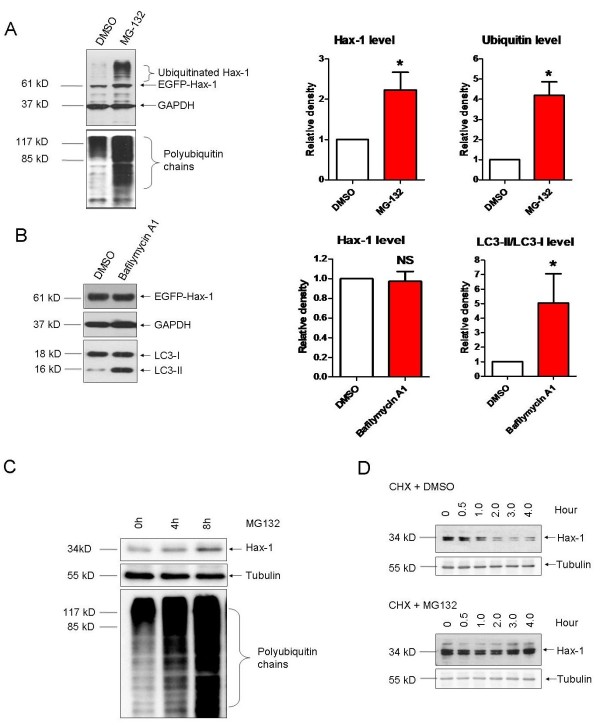
**Proteasomal degradation of Hax-1. A** and **B**. H1299 cells were transiently transfected with EGFP-Hax-1. Forty-eight hours later, cells were treated with MG132 (1 μM) for 3 hours (A) or Bafilomycin A1 (10 nM) for 18 hours (B). Cells were then harvested and immunoblotted using anti-GFP, ubiquitin, LC3 or GAPDH antibody. Detections of polyubiquitin (A, left, lower panel) and LC3-II (B, left, lower panel) levels in input were used to indicate polyubiquitination levels (A) and autophagy levels (B). Data from three independent experiments were quantified (means ± S.E.M., *p < 0.05, one-way ANOVA). **C**. Time dependent increase of Hax-1 levels by the proteasomal inhibition (Upper panel). Increased levels of total ubiquitin were served as positive controls for MG132 treatment. Tubulin was served as loading control. **D**. H1299 cells were treated with CHX with or without MG132 for chase experiments.

Proteasome and autophagy systems are two main pathways for protein degradation. Here we tested which pathway is involved in the fast-turnover of Hax-1. Cells were treated with MG132, a proteasome inhibitor, or Bafilomycin A1, an autophagy inhibitor. The level of EGFP-Hax-1 increased in cells treated with MG132 for 3 hours (Figure 2A), whereas in cells treated with Bafilomycin A1 the protein level remained unchanged up to 18 hours (Figure 2B). These data suggest that Hax-1 is mainly degraded by the proteasome, but not by autophagy-lysosome pathway. A time-dependent increase in endogenous Hax-1 level was also observed in cells treated with MG132 (Figure 2C). We next examined the turnover of endogenous Hax-1 in the presence of MG132 using CHX chase experiments. In the presence of MG132, endogenous Hax-1 was not observed to be degraded within 4 hours, however, in the absence of MG132, it was rapidly degraded after two hours (Figure 2D).

### Hax-1 conjugation with K48-linked ubiquitin chains is dependent on the PEST sequence

**Figure 3:**
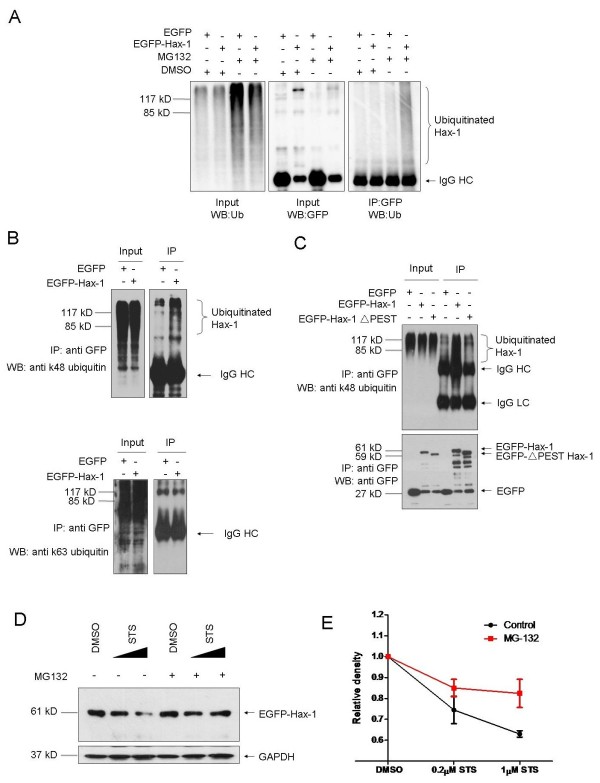
**K48-linked ubiquitiniation of Hax-1 enhanced proteasomal degradation of Hax-1 during apoptosis.A**. H1299 cells were transfected with EGFP or EGFP-Hax-1. Forty-eight hours later, cells were treated with or without MG132 (1 μM) for 12 hours. Cell lysates were then subjected to immunoprecipitation with anti-GFP antibodies and the immunoprecipitants were detected with total ubiquitin antibody. **B**. Similar experiments as A were performed using specific anti-K48 or -K63 ubiquitin antibodies. **C**. H1299 cells were transfected with EGFP or EGFP-Hax-1 or EGFP-ΔPEST Hax-1 expression constructs. Forty-eight hours later, cells were treated with MG132 (1 μM) for 12 hours. Cell lysates were then immunoprecipitated with anti-GFP antibodies and detected with anti-K48 ubiquitin antibodies. **D**. EGFP-Hax-1 transfected H1299 cells were treated with DMSO or increased levels of STS (0.2-1 μmol) in the absence or presence of MG132 for 3 hours. Cells were then harvested and subjected to immunoblot analysis with anti-GFP antibody. **E**. Quantitative analyses of Hax-1 degradation upon STS treatment through three independent experiments were shown.

We have shown that Hax-1 is degraded by the proteasome. Usually, the proteasomal degradation process requires polyubiquitination of the substrates [21]. We therefore tested if Hax-1 is ubiquitinated and if yes, what kind of ubiquitin conjugation is involved in the degradation of Hax-1. Enhanced ubiquitination of Hax-1 was observed in the presence of MG132 than that in the absence of MG132 (Figure 3A) as revealed by co-immunoprecipitation experiments. Then, we examined the polyubiquitin of Hax-1 with two specific antibodies which recognize K48- or K63-linked ubiquitin, respectively. Increased polyubiquitination of Hax-1 was detected with an antibody specific to K48-linked polyubiquitin, but not with that to K63-linked polyubiquitin (Figure 3B), suggesting that Hax-1 is mainly conjugated by the K48-linked ubiquitin chains. We next evaluated if the PEST sequence affects Hax-1 polyubiquitination. We found that the deletion of the PEST sequence in Hax-1 greatly decreased its polyubiquitination (Figure 3C), suggesting that the PEST sequence in Hax-1 is necessary for its ubiquitination.

### Increased degradation of Hax-1 during apoptosis

As Hax-1 is known to be an anti-apoptotic protein, we hypothesized whether its degradation is regulated under apoptosis. We transfected H1299 cells with EGFP-Hax-1 and treated them with DMSO or staurosporine (STS), an inducer of apoptosis. In the absence of MG132, the amounts of Hax-1 protein decreased with increasing concentration of STS, however, in the presence of MG132, the trend was largely attenuated (Figure 3D and E), suggesting an accelerated degradation of Hax-1 by the proteasome under apoptosis.

### ΔPEST Hax-1 mutant attenuated STS-induced cell death

**Figure 4:**
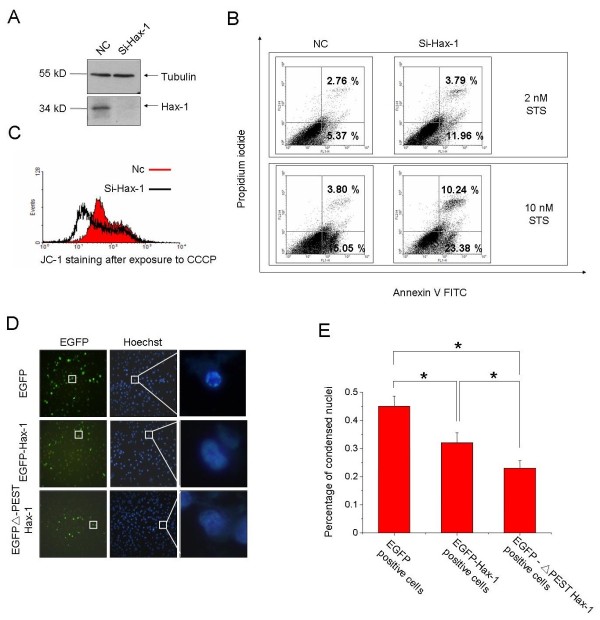
**ΔPEST Hax-1 strongly attenuates apoptosis. A**. Knockdown efficiency of Hax-1 siRNA used in this study. **B**. H1299 cells were transfected with negative control (NC) or siRNA against Hax-1 and then treated with different doses of STS (2–10 nM) for 2 hours. Cells were double stained with annexin V-FITC and PI followed by FACS analyses. **C**. H1299 cells were transfected with NC or siRNA against Hax-1 and then treated with CCCP (10 μM) for 30 minutes. After CCCP treatment, cells were stained with a JC-1 mitochondrial membrane potential detection kit. **D**. H1299 cells were transfected with EGFP or EGFP-Hax-1 or EGFP-ΔPEST Hax-1. Forty-eight hours later, cells were treated with STS (20 nM) for 12 hours and stained with Hoechst 33342. **E**. Quantitative analysis of condensed nuclei from D with three independent experiments was performed; random field of 250 cells were counted. (means ± S.E.M., *p < 0.05, one-way ANOVA).

As overexpression of Hax-1 has been shown to have an anti-apoptotic effect and also regulates mitochondria membrane potential [10], we examined the effects of knockdown of Hax-1 on STS-induced apoptosis. The efficacy of the siRNA against Hax-1 was evaluated (Figure 4A). STS induced significantly higher level of apoptosis in those cells in which Hax-1 levels were knocked down as compared to control cells. This increase in apoptosis also elevated with increased STS dosage (Figure 4B). Using JC-1 (a lipophilic, cationic dye that selectively enters into mitochondria and reversibly changes its color from green to red as the membrane potential increases) staining, we found that mitochondrial potential was also greatly decreased in Hax-1 knockdown cells than in control cells upon CCCP (Carbonyl cyanide m-chlorophenyl hydrazone, an inducer to cause mitochondrial permeability transition) treatment (Figure 4C). These data indicate that Hax-1 is important for cells against apoptotic stress or mitochondrial damage. We next transfected cells with WT Hax-1 or ΔPEST Hax-1 and then treated cells with STS. Fewer condensed nuclei were observed in EGFP-ΔPEST Hax-1 expressing cells than in EGFP-Hax-1 expressing cells (Figure 4D and E), suggesting that deletion of PEST sequence may increase Hax-1 stability, causing more resistance to STS-induced apoptosis.

## Discussion

Hax-1 transcript levels in mouse kidney, testis, and liver have previously been found to not directly correlate with detected protein levels [22]. Similar phenomenon has also been observed in rat tissues [23]. Two hypotheses to explain the different levels of mRNA compared to protein are that either high amounts of the Hax-1 transcript do not translate into proteins or that the protein degradation rate of Hax-1 is considerably high [24]. Here, we provide clear evidence showing that Hax-1 protein is indeed turned over at a fast rate in a proteosome dependent manner. It is important to note that, Hax-1 exists as many as 7 alternative splicing forms [16,23], and these splicing variants may play important roles in development or tumor formation. For example, the internal deletions in variants vII, vIV and vVI result in removal of BH domains and changes in PEST domain from variants I (the full length 278aa form which is investigated in this paper) [23]. It is therefore possible that these variant forms of Hax-1, because of its impairment in PEST degradation signal, is more stable than its dominant form variant I. The population of cells bearing an up-regulation of these variants shows enhanced protective roles in tissues or more oncogenic activity, as evidenced in tumors [16].

Polyubiquitination is required for the protein degradation by the proteasome [21]. Ubiquitin molecules, which form ubiquitin chains to a protein, are covalently linked to each other between a lysine site (K11, K29, K48 or K63) of the previous ubiquitin and the carboxy-terminal glycine of a new ubiquitin. K48-linked polyubiquitination of a protein usually mediates its degradation by the proteasome, however, K63-linked polyubiquitination is most likely to play roles in translation, endocytosis and other functions [25-27]. In the present report, we demonstrate that Hax-1 is ubiquitinated via K48-linked ubiquitin chains. The ubiquitination of Hax-1 is largely dependent on its PEST sequence. In many short-lived proteins, the PEST sequence serves as a signal sequence to drive their proteolysis or rapid degradation [17]. In some cases, ubiquitination of proteins depends upon their PEST sequence [19]. Here, we found that deletion of the PEST sequence results in much less ubiquitination of Hax-1, thereby increasing its stability. It is therefore possible that the PEST sequence in Hax-1 is responsible for its proper folding to be conjugated with the ubiquitin chains. The PEST sequence is also reported to be a motif that is involved in protein modification. For example, phosphorylation of a PEST sequence by casein kinase II (CKII) appears to promote the degradation of IκBα [28]. Also, a PEST-like sequence has been shown to mediate phosphorylation and efficient ubiquitination of yeast uracil permease [29]. Further studies to identify if the PEST sequence in Hax-1 is phosphorylated and if this modification affects Hax-1 stability will be of help to explore the exact role of the PEST sequence in Hax-1.

Hax-1 is structurally similar to Bcl-2 for its BH domains and TM domain. However, Hax-1 is less stable compared to other Bcl-2 family proteins [30,31]. It was reported that Hax-1 is rapidly cleaved by caspase 3 [32], HtrA2 [10] or Granzyme B [33] during cell death. It is therefore possible that these enzymes contribute to Hax-1 degradation in apoptosis. As Hax-1 is a short-lived protein and also degraded by the proteasome, it suggests that the proteasomal degradation of Hax-1 highly regulates Hax-1 levels in normal conditions. Knockdown of pleiotropic human prohibitin 2 in HeLa cells results in caspase-dependent apoptosis through down-regulation of Hax-1 [34]. Here, we report that, in addition to protease cleavage, the proteasomal degradation is also an important post-translational regulation for Hax-1 during apoptosis (Figure 3D and E). When the PEST sequence is abolished, Hax-1 is shown to convey increased resistance to cell death. Taken together, these data suggest that Hax-1 may be rapidly subjected to proteolysis in response to cellular stresses, resulting in a decrease in its protein level and hence loss of its protective activity.

## Conclusions

In summary, our study demonstrates that Hax-1 is rapidly degraded by the proteasome in a PEST sequence dependent manner. During apoptosis, degradation of Hax-1 is enhanced whereas expression of ΔPEST mutant of Hax-1 protects cells against apoptotic stimulation.

## Methods

### Cell culture, transfections and drug treatments

N2a and H1299 cells were grown in Dulbecco’s Modified Eagle’s Medium (DMEM, GIBCO) containing 10 % fetal calf serum with 100 μg/ml penicillin and 100 μg/ml streptomycin. Transfections were performed using Lipofectamine 2000 (Invitrogen) according to the manufacturer’s instructions. In order to ensure equal transfection efficiency, master mix of the same plasmids were made and aliquot to each well, we double check the equal expression of EGFP-Hax-1 through fluoresce microscopy before drug treatment (by deem to lowest exposure). Hoechst 33342, DAPI, STS (Staurosporine), Bafilomycin A1, Annexin V, PI (Propidium iodide) and CHX (cycloheximide) were purchased from Sigma. MG132 was obtained from Calbiochem.

### Plasmids

The Hax-1 related constructs were described previously [35]. A PEST sequence deletion mutant was created using the following primers: 5’-ACCAAGATCACTAAACCA-3’ and 5’-CTGTAGAACCGGGCCAAG-3’.

### siRNAs

35 pmoles of each siRNA were transfected using Oligofectamine, according to the manufacturer’s instructions (Invitrogen). Oligonucleotides were purchased from GenePharma (Shanghai, China) and had the following sequences:

si *Hax-1* sense: 5’-AACCAGAGAGGACAAUGAUCUdTdT-3’.

si *Hax-1* antisense: 5’-AGAUCAUUGUCCUCUCUGGUUdTdT-3’.

si Control sense: 5'-UUCUCCGAACGUGUCACGUdTdT-3'.

si Control antisense: 5'-ACGUGACACGUUCGGAGAAdTdT-3'.

### Immunoblot analysis and antibodies

Cell extracts were lysed in 1 × RIPA lysis buffer (25 mM Tris–HCl, pH 7.6, 150 mM NaCl, 1 % NP-40 and 1 % sodium deoxycholate) in the presence of protease inhibitor cocktail (Roche). Approximately 20 μg of cell lysates was separated on SDS-PAGE and transferred onto a PVDF membrane (Millipore). Immunoblot analyses were carried out with the following primary antibodies: anti-Bcl-2 (Abcam), anti-Bcl-xL (Cell Signaling Technology), anti-GAPDH (Chemicon), anti-GFP (Santa Cruz Biotechnology), anti-LC3 (Novas), anti-Tubulin (Merck Chemicals), anti-Hax-1 (BD Biosciences), anti-Flag (Sigma), anti-ubiquitin (Santa Cruz Biotechnology), anti-K48-ubiquitin (Millipore) and anti-K63-ubiquitin (Millipore). The secondary antibodies, i.e., sheep anti-mouse IgG-HRP or anti-rabbit IgG-HRP, were from Amersham Pharmacia Biotech. The proteins were visualized using an ECL detection kit (Amersham Pharmacia Biotech).

### Immunoprecipitation

Cells transfected with the indicated plasmids were collected 48 hrs after transfection and were lysed in TSPI buffer containing 50 mM Tris–HCl, pH 7.5, 150 mM sodium chloride, 1 mM EDTA and 1 % NP-40 supplemented with complete mini protease inhibitor cocktail (Roche). Cellular debris was removed by centrifugation at 12,000 g for 30 minutes at 4°C. The supernatants were incubated with anti-GFP antibodies overnight at 4°C. After incubation, protein G Sepharose (Roche) was used for precipitation. The beads were washed with TSPI buffer four times and then eluted with SDS sample buffer for immunoblot analysis.

### Statistical analysis

Densitometric analysis of immunoblots from three independent experiments was performed using ImageJ windows version. The data were analyzed using windows version of Origin 6.0 (Originlab) or Prism 5 (Graphpad softwere). The pictures in Figure 1A were draw using DOG 1.0 [36].

## Abbreviations

CHX, Cycloheximide; Hax-1, HS-1-associated protein X-1; PCR, Polymerase Chain Reaction; SDS-PAGE, Sodium Dodecyl Sulfate-Polyacrylamide Gel Electrophoresis; siRNA, Small interfering RNA; STS, Staurosporine; CCCP, Carbonyl Cyanide m-Chlorophenyl hydrazone.

## Competing interests

The authors declare no conflict of interest in this paper.

## Authors’ contributions

BL performed most experiments in plasmid construction, immunoblot analyses, immunoprecipitations, cell culture, transfections, drug treatments, siRNAs, statistical analysis and drafted the manuscript; QH performed parts of experiments in cell transfections, immunoprecipitationss; RX performed part of plasmid construction; HR performed part of cell culture and transfections; EF performed part of plasmid construction; DC performed part of cell cultures; GW conceived of the study, participated in its design and coordination and revised manuscript. All authors read and approved the final manuscript.
